# Assessment of Carotid Body Tumors by Superb Microvascular Imaging of Feeding Arteries During Preoperative Evaluation

**DOI:** 10.3389/fsurg.2022.816768

**Published:** 2022-04-26

**Authors:** Luying Gao, Xiaoyan Zhang, Yuxin Jiang, Hongyan Wang, Yuehong Zheng, Wanying Li, Jianchu Li, Bo Zhang

**Affiliations:** ^1^Department of Ultrasound, State Key Laboratory of Complex Severe and Rare Diseases, Peking Union Medical College Hospital, Chinese Academy of Medical Sciences and Peking Union Medical College, Beijing, China; ^2^Department of Vascular Surgery, State Key Laboratory of Complex Severe and Rare Diseases, Peking Union Medical College Hospital, Chinese Academy of Medical Sciences and Peking Union Medical College, Beijing, China; ^3^Department of Ultrasound, China-Japan Friendship Hospital, Beijing, China

**Keywords:** carotid body tumor, superb microvascular imaging, ultrasound, color Doppler flow imaging, color Doppler

## Abstract

**Purpose:**

Superb microvascular imaging (SMI) has led to new advances in vascular imaging applications. This study aimed to explore the blood supply and feeding arteries of carotid body tumors (CBTs) on SMI to improve the accuracy of information available to surgeons.

**Methods:**

Twenty-six CBT lesions were subjected to color Doppler flow imaging (CDFI) and SMI and were later confirmed by pathology. The blood flow patterns and feeding arteries of the CBTs on CDFI and SMI were graded and compared.

**Results:**

The feeding arteries of two CBT lesions, which were not visible on CDFI, were identified as the internal carotid artery (ICA) on SMI. The feeding arteries of three CBTs were judged to stem from both the ICA and the external carotid artery (ECA) (MIX) based on SMI compared to the ICA or ECA on CDFI. We classified the feeding arteries of CBTs as originating from the ICA or others (including the ECA and MIX). One hundred percent (3/3) of the CBT lesions stemming from the ICA had Adler I or Adler II blood flow patterns, and 100% (23/23) of the CBT lesions stemming from other arteries had Adler II or Adler III blood flow patterns. Higher Adler categories were assigned based on SMI than CDFI (*P* < 0.001).

**Conclusion:**

SMI may be superior to CDFI in detecting the vascularity of CBTs, and SMI revealed more potential feeding arteries of CBTs than CDFI. CBTs originating from the ICA are less vascular than those originating from the ECA.

## Introduction

Carotid body tumors (CBTs) are rare non-chromaffin paragangliomas involving the carotid body chemoreceptor and represent more than 50% of head and neck paragangliomas (1, 2). Because CBTs have a malignant tendency for local or distant metastasis (3), surgical excision is advisable (4). Due to the slow growth of CBTs, detection usually requires a long time. As a result, most patients already exhibit Shamblin grade II or III at the time of surgery, which can damage the internal carotid artery (ICA) and external carotid artery (ECA), with incidences of 11% and 13–33%, respectively (3, 4). Determining the anatomical relationship between the CBT and carotid arteries is important before the operation to reduce surgical complications and to judge whether the external carotid artery needs to be clamped during the operation. A bloodless operative field can be achieved to reduce operative morbidity. Thus, reliable and effective imaging modalities for recognizing the relationship between carotid arteries and CBTs are crucial.

Due to the hypervascularity of CBTs and their proximity to vascular and nervous structures, fine-needle aspiration biopsy (FNAB) is not appropriate (1, 5). Therefore, diagnostic imaging modalities are essential for the diagnosis of CBTs. Ultrasound (US) has been widely used with the development of color Doppler flow imaging (CDFI) to enhance CBT characterization. Tumors are highly dependent on angiogenesis. However, due to clutter and overflow on CDFI, it is difficult to obtain full and accurate vascular information on US. Superb microvascular imaging (SMI) uses a new adaptive algorithm to identify and remove tissue motion and reveal true blood flow. It has led to advances in vascular imaging, facilitating the overall detection of vascularity of small or microflow states without the use of contrast media. SMI has been demonstrated to be effective in identifying microflow in several clinical settings, such as breast cancers and thyroid nodules, by our team and other researchers (6–8). To date, no clinical research has been reported on SMI in the evaluation of CBT vascularity. The purpose of this study was to better explore the blood supply and feeding arteries of CBTs on SMI to provide more accurate information for surgery.

## Materials and Methods

### Patients

Between September 2017 and December 2019, 21 patients with 26 CBT lesions were referred to our center for surgical treatment of lateral neck masses. Within this cohort of patients, those who met the following criteria were included in the study: (1) patients who underwent conventional US and SMI for lateral neck masses before surgical excision at our center; (2) patients who were treated with surgical resection following a standard procedure; and (3) patients with postoperative workup that confirmed the CBT diagnosis.

### US Examination

All US examinations were performed with Aplio 500 (Canon Medical Systems, Tokyo, Japan) devices equipped with an L14-5 linear array transducer. US was performed by two radiologists who were experienced in ultrasonography (GLY and ZXY, with more than 5 years of US experience) and were blinded to the patients' clinical data. In cases of a discrepancy between the two radiologists, a consensus was reached after discussion.

The lesion size, location, number, margin, echogenicity, and structure were evaluated using conventional B-mode US. The vascularity and relationship of lesions with carotid bifurcation, ICA and ECA were evaluated by CDFI and SMI. For SMI, the probe was placed above the lesion, and the sampling range was adjusted to cover the entire lesion and surrounding tissue with a mechanical index of 1.5, a low-speed scale of 1.0–2.0 cm/s, a frame rate of 50–60 fps, and a dynamic range of 55–60 dB. The SMI parameters for each CBT lesion were regulated to best reveal the vascularity with minimal noise, and two SMI modes were available: monochrome and color. A double screen function that allowed grayscale imaging and SMI visualization at the same time was used when vessels were located. The data were stored as images and videos. The feeding arteries of the CBT lesions were observed when the video was repeatedly played back. The vascularity of the CBTs was classified into 4 patterns (9): Adler 0, with no substantial vascularity (defined as no perivascular flow or dotted perinodular flow on <25% of the lesion circumference and without any internal flow); Adler I, with peripheral vascularity (defined as blood flow surrounding >25% of the lesion circumference without any internal flow); Adler II, with mixed vascularity (defined as the presence of any intranodular flow and peripheral flow on >25% of the lesion circumference); and Adler III, with intranodular vascularity (defined as exclusively internal flow, including vessels penetrating the lesion and isolated center vessels).

### Statistical Analysis

The Shapiro–Wilk test was used to determine the presence of a normal distribution. For parametric data, an unpaired *t*-test was used to evaluate differences between the two groups. For non-parametric data, differences between groups were analyzed using a Mann–Whitney *U*-test. The chi-square test with Yates' correction and Fisher's exact test were used to compare categorical variables. Consistency was assessed using the kappa value, which was assigned as follows: 0–0.25, slight agreement; 0.25–0.49, fair agreement; 0.50–0.69, moderate agreement; 0.70–0.89, excellent agreement; and 0.90–1.00 almost perfect agreement. A value of *P* < 0.05 was considered statistically significant. Statistical analyses were performed with SPSS software (Version 19.0, SPSS Chicago, IL, USA).

## Results

### Clinical and US Characteristics of CBT Lesions

A total of 21 patients with 26 CBT lesions were included in the study. The patients included 7 (26.9%) females and 19 (73.1%) males with a median age of 43.2 years (range, 31–63 years). Sixteen patients had a single lesion, and five patients had bilateral lesions.

All the CBT lesions (100%) were detected by US. The mean size of the CBT lesions on US was 3.46 cm (range, 1.5–5.0 cm). The echogenicity of the lesions was determined to be solid in 23 (88.5%) lesions and mixed structure in 3 (11.5%) lesions. Furthermore, all solid parts of the lesions (100%) were hypoechoic with well-circumscribed margins and were located in the carotid bifurcation, leading to the separation of the ICA and ECA (Table 1).

**Table 1:** Clinical and US characteristics of CBT lesions.

	**CBT**
No. of nodules	26
Age (y; mean, range)	43.2 (31–63)
Sex (male: female)	19:7
Size (cm; mean, range)	3.46 (1.5–5.0)
US features	
Composition	
Solid	23 (92%)
Partially cystic	3 (8%)
Echogenicity	
Hypoechoic	26 (100%)
Margin	
Irregular	0
Well-circumscribed	26 (100%)

*CBT, carotid body tumor*.

### Blood Flow Characteristics and Differences Between SMI and CDFI

**Figure 1:**
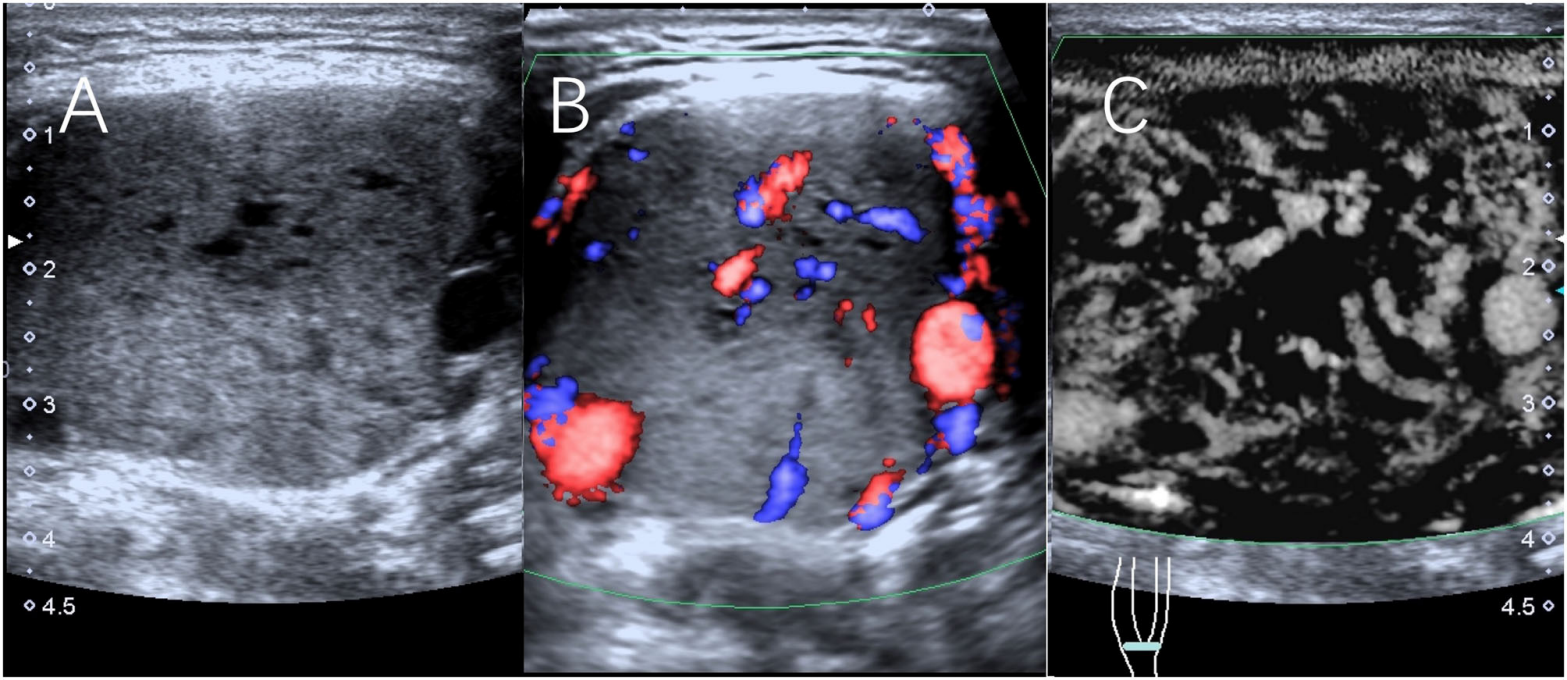
Scans from a 32-year-old man with a right CBT lesion measuring 4.4 cm. **(A)** Grayscale sonography showing the lesion. **(B)** The lesion was classified as having Adler II vascularity on CDFI. **(C)** The lesion was classified as having Adler III vascularity on SMI.

**Figure 2:**
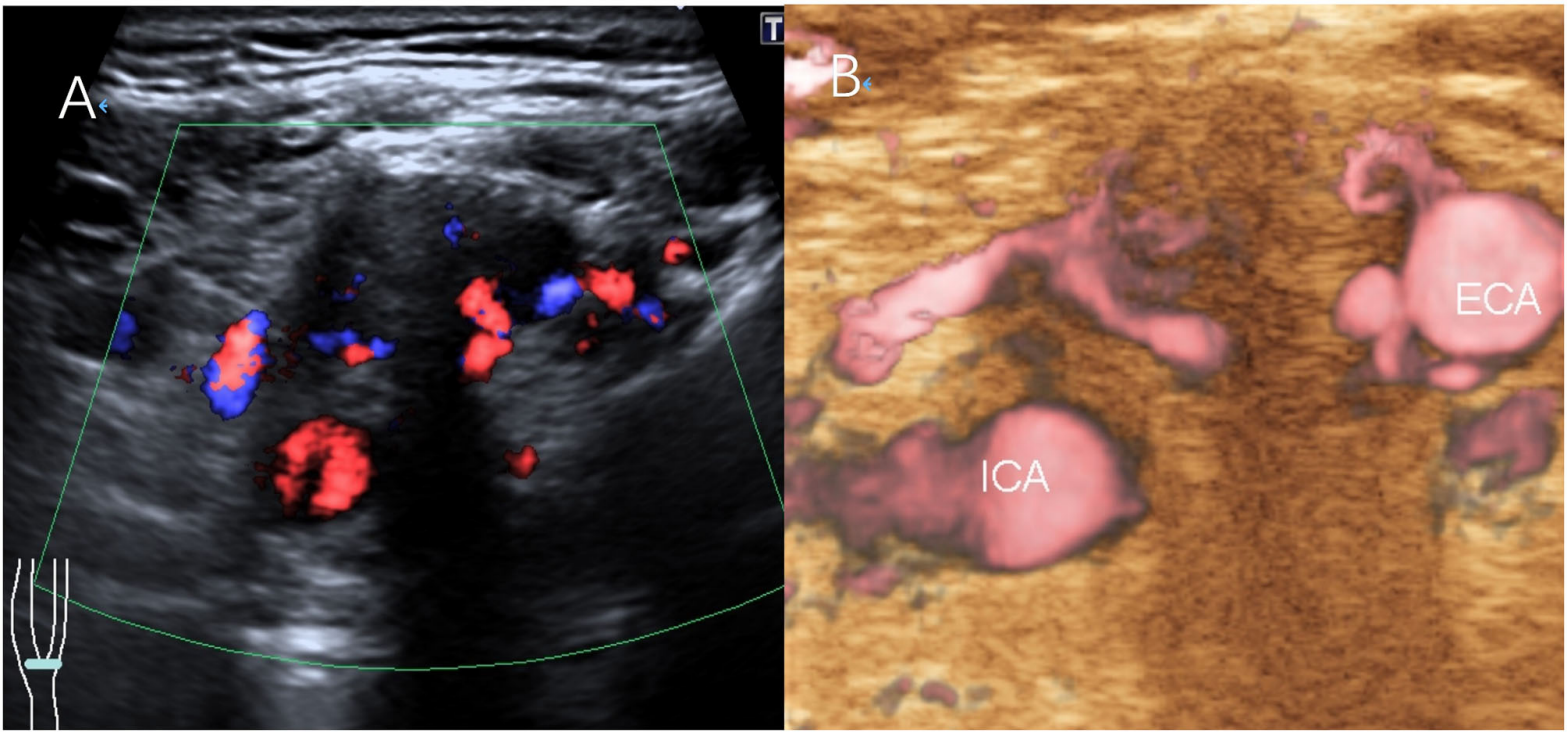
Scans from a 38-year-old man with a right CBT lesion measuring 3.8 cm. **(A)** CDFI of the lesion. **(B)** The feeding artery of the CBT on SMI.

The distributions of the blood flow patterns of all the lesions were compared between CDFI and SMI. The blood flow categories of the 26 lesions differed between the two methods (Table 2). The Adler category was upgraded with SMI compared to CDFI (*P* < 0.001). On CDFI, 23.1% (6/26) of the CBT lesions had Adler I blood flow patterns, 57.7% (15/26) had Adler II blood flow patterns, 11.5% (3/26) had Adler III blood flow patterns, and 7.7% (2/26) had Adler 0 blood flow patterns. On SMI, 7.7% (2/26) of the CBT lesions had Adler I blood flow patterns, 30.8% (8/26) had Adler II blood flow patterns, and 61.5% (16/26) had Adler III blood flow patterns. On SMI, the pattern of two CBTs changed from Adler 0 to Adler I, the pattern of six CBTs changed from Adler I to Adler II or III, and the pattern of 10 CBTs changed from Adler II to Adler III (Figures 1, 2).

**Table 2:** Blood flow characteristics and differences between SMI and CDFI.

**SMI**					
**CDFI**	**I**	**II**	**III**	**Total**	***P***
0	2	0	0	2 (7.7%)	0.008
I	0	3	3	6 (23.1%)	
II	0	5	10	15 (57.7%)	
III	0	0	3	3 (11.5%)	
Total	2 (7.7%)	8 (30.8%)	16 (61.5%)	26	

*SMI, superb microvascular imaging; CDFI, color Doppler flow imaging*.

The CBTs were classified according to Shamblin criteria: type 1 in 9 cases (34.6%), type 2 in 11 cases (42.3%), and type 3 in 6 cases (23.1%). For the Shamblin type 3 CBT lesions, no lesions had Adler I blood flow patterns, and more lesions had Adler II/III blood flow patterns than the Shamblin type 1/2 CBT lesions, but the difference was not statistically significant (6/6, 100% vs. 18/20, 90%, *P* = 0.30) (Supplementary Table 1). We further analyzed the correlation between intraoperative blood loss and Shamblin type. Blood loss was higher in the patients with Shamblin type 3 lesions than in those with Shamblin type 1/2 lesions (320.0 vs. 62.8 ml, *P* = 0.001).

All the patients underwent surgical excision, and the lesions were confirmed as benign by pathology. Histologically, tumor cells had local invasiveness in only one case (1/26, 3.8%). The lesions had Adler III blood flow patterns on SMI compared to Adler II blood flow patterns determined by CDFI. Immunohistochemical staining of the tumors showed that the positive rates of chromogranin A (CgA) and synaptophysin (Syn) were 21/23 and 13/13, respectively, which showed varying numbers of brown granules in the cytoplasm of tumor cells. The positive rate of S-100 protein was 23/24. A few positive cells were scattered among tumor cells, which were considered supporting cells.

### Characteristics of Feeding Arteries and Their Differences Between SMI and CDFI

On CDFI, the feeding artery was judged to stem from the ICA in 11.5% (3 of 26) of the CBTs, from the ECA in 26.9% (7 of 26) of the CBTs, and from both the ICA and ECA in 53.8% (14 of 26) of the CBTs. The feeding artery of two CBT lesions (7.7%) had no color flow on CDFI. On SMI, the feeding artery was judged to stem from the ICA in 11.5% (3 of 26) of the CBTs, from the ECA in 23.1% (6 of 26) of the CBTs, and from both the ICA and ECA in 65.4% (17 of 26) of the CBTs. On SMI, the judgment changed from the ICA to both the ICA and ECA for two CBTs and from the ECA to both the ICA and ECA for one CBT. The feeding arteries of the two CBT lesions that were not observed on CDFI were determined to be the ICA on SMI. The consistency of SMI and CDFI was assessed using kappa values and reached a moderate level with a value of 0.66 (Table 3).

**Table 3:** Characteristics of feeding arteries and their differences between SMI and CDFI.

**SMI**					
**CDFI**	**ICA**	**ECA**	**MIX**	**Total**	***P***
Unable to	2	0	0	2 (7.7%)	<0.001
judge					
ICA	1	0	2	3 (11.5%)	
ECA	0	6	1	7 (26.9%)	
MIX	0	0	14	14 (53.8%)	
Total	3 (11.5%)	6 (23.1%)	17 (65.4%)	26	

*SMI, superb microvascular imaging; CDFI, color Doppler flow imaging; ECA, external carotid artery; ICA, internal carotid artery*.

We classified the feeding artery of CBTs as originating from the ICA and others [including the ECA and both the ECA and ICA (MIX)]. Of all the lesions, three lesions stemmed from the ICA, and 23 lesions stemmed from the ECA or MIX. Based on SMI, we calculated the classification of blood flow in lesions stemming from the ICA and others. Among the CBT lesions stemming from the ECA or MIX, 30.4% (7/23) and 69.6% (16/23) had Adler II and Adler III blood flow patterns, respectively, while among the CBT lesions stemming from the ICA, 66.7% (2/3) and 33.3% (1/3) had Adler I and Adler II blood flow patterns, respectively (Table 4).

**Table 4:** Blood flow characteristics of CBTs originating from the ICA and other arteries (including the ECA and MIX).

**Adler**				
**Feeding artery**	**I**	**II**	**III**	***P***
ICA	2 (66.7%)	1 (33.3%)	0	<0.001
ECA and MIX	0	7 (30.4%)	16 (69.6%)	

*CBT, carotid body tumor; ECA, external carotid artery; ICA, internal carotid artery*.

A computed tomography (CT) scan was performed in all the patients showing a solid tumor. With this scan, the feeding artery was found to stem from the ICA in 7.7% (2 of 26) of the CBTs, from the ECA in 42.3% (11 of 26) of the CBTs, and from both the ICA and ECA in 46.2% (12 of 26) of the CBTs. The feeding artery of one CBT lesion (3.8%) failed to be displayed. The consistency of SMI and CT was assessed using kappa values and reached a level with a value of 0.43 (Supplementary Table 2).

Embolization was performed before surgery in one case only. In this case, CTA and CDFI showed that the vascular supply was from the ECA. On SMI, the feeding artery was found to stem from the ECA and ICA. Catheter angiography confirmed that the vascular supply to the CBT arose from the ECA and ICA.

## Discussion

SMI allows radiologists to visualize the microvascular patterns of lesions in detail without the additional use of a contrast agent (10). A previous study showed that contrast-enhanced US allowed easy identification of the collateral vessels of cervical body tumor masses (11). Previous studies also reported that SMI provided a better depiction of vessel branching details than CDFI in thyroid nodules (12). In our study, we found that SMI is superior to CDFI in assessing the blood flow of CBTs. On SMI, the judgment of the blood flow pattern of two CBTs changed from Adler 0 to Adler I, the pattern of six CBTs changed from Adler I to Adler II or III, and the pattern of ten CBTs changed from Adler II to Adler III. This may have occurred because some feeding vessels were too small to be observed by CDFI. CDFI is unable to detect low-velocity blood flow because of the presence of extraneous Doppler signals due to nearby structures. SMI can show lower-velocity blood flow because it can analyze clutter motion and uses a new adaptive algorithm to identify and remove tissue motion and reveal true blood flow. Because there is almost no angle dependence, clutter, or overflow at lower scales, SMI shows much more complete and accurate vascular branches.

We showed that SMI was superior to CDFI in detecting the feeding artery of CBTs. Two lesions (7.7%) that stemmed from the ICA were not detected with CDFI, and three lesions (11.5%) that stemmed from both the ICA and ECA were not detected accurately with CDFI. Catheter angiography was performed before surgery in one case only. In this case, the lesion stemming from both the ICA and ECA was not detected accurately with CDFI and CT. Vascular supply arises from the ECA and ICA on SMI, which was confirmed by catheter angiography. CDFI relies on longitudinal and transverse section imaging and shows the feeding artery in a single plane; therefore, it cannot fully reflect vascular spatial heterogeneity. Identification of the feeding artery has significance for both preoperative diagnosis and successful resection. With preoperative selective embolization or intraoperative clamping of the carotid artery feeding branches, a bloodless operative field could be achieved (13, 14). For tumors close to the carotid artery, forcibly separating the tumor may cause the blood vessel to rupture, resulting in heavy bleeding. CBTs that invade the ICA and ECA are often difficult to separate from the ECA, and the proximal end of the ECA can be clamped before the tumor is removed.

We classified the feeding arteries of the CBTs as originating from the ICA and others (including the ECA and MIX) and found that the vascularity was different in the CBTs stemming from the ICA vs. those stemming from others. All the CBTs originating from the ICA had an Adler I/II pattern; however, only 30.4% of the CBTs originating from the ECA and MIX had an Adler II pattern, and most of the CBTs (69.6%) originating from the ECA had an Adler III pattern. The results showed that the CBTs originating mainly from the ICA have less vascularity than those originating from the ECA. The immunohistochemical results showed that 21/23 and 13/13 of the tumor cells showed positive reactions of CgA and Syn, suggesting that some tumor cells have potential endocrine functions.

There are several limitations to our study. First, the vascularity patterns were not analyzed separately for malignant and benign lesions because all the lesions were benign CBTs. The scope of this study was limited by the lack of malignant CBTs and neurogenic tumors, such as schwannomas. Second, our study included 26 lesions, and future studies involving more subjects may produce more accurate results. Third, the lack of pathological correlation is a significant limitation of our work. Fourth, the results may have been affected by selection bias, as only patients who underwent both US and surgery were enrolled in the study.

## Conclusions

We showed that compared to CDFI, SMI may be used to better investigate the vascularity of CBTs and that SMI revealed more potential feeding arteries of lesions than CDFI. In addition, CBTs originating mainly from the ICA have less vascularity than those originating from the ECA or MIX. We expect that with the application of SMI, this approach could become an invaluable tool for the diagnosis and preoperative workup of CBTs.

## Data Availability Statement

The raw data supporting the conclusions of this article will be made available by the authors, without undue reservation.

## Ethics Statement

The studies involving human participants were reviewed and approved by Peking Union Medical College Hospital. The patients/participants provided their written informed consent to participate in this study. Written informed consent was obtained from the individual(s) for the publication of any potentially identifiable images or data included in this article.

## Author Contributions

JL and BZ conceived of and designed the study. XZ, HW, LG, WL, and YZ participated in the study design, data verification, image quality verification, and selection and collection of samples. LG and XZ performed the analysis and prepared all the figures and tables. LG was the main contributor to the writing of the manuscript. YJ edited the manuscript. All the authors read and approved the final manuscript.

## Funding

This work was supported by the National Natural Science Foundation of China (61971448) and the CAMS Innovation Fund for Medical Sciences (2016-I2M-1-011).

## Conflict of Interest

The authors declare that the research was conducted in the absence of any commercial or financial relationships that could be construed as a potential conflict of interest.

## Publisher's Note

All claims expressed in this article are solely those of the authors and do not necessarily represent those of their affiliated organizations, or those of the publisher, the editors and the reviewers. Any product that may be evaluated in this article, or claim that may be made by its manufacturer, is not guaranteed or endorsed by the publisher.

## Abbreviations

CBT: carotid body tumor
CDFI: color Doppler flow imaging
SMI: superb microvascular imaging
US: ultrasound
ECA: external carotid artery
ICA: internal carotid artery
CEUS: contrast-enhanced ultrasound.
