# Supplementary material for: Assessment of Carotid Body Tumors by Superb Microvascular Imaging of Feeding Arteries During Preoperative Evaluation
Source: Front Surg. 2022 Apr 26;9:816768. doi: 10.3389/fsurg.2022.816768 (PMC9090302; doi:10.3389/fsurg.2022.816768)
Supplement: Supplementary file 1 [file Data_Sheet_1.docx]

**Supplemental Table 1.** Correlation between Adler type of SMI and Shamblin criteria

|  | SMI | | |  |  |
| --- | --- | --- | --- | --- | --- |
| Shamblin type | I | II | III | Total | P |
| 1 | 1 | 2 | 6 | 9 (34.6%) | 0.29 |
| 2 | 1 | 2 | 8 | 11 (42.3%) |  |
| 3 | 0 | 4 | 2 | 6 (23.1%) |  |
| Total | 2 (7.7%) | 8 (30.8%) | 16 (61.5%) | 26 |  |

SMI, superb microvascular imaging

**Supplemental Table 2.** Characteristics of feeding arteries and their differences between SMI and CT

|  | SMI | | | |  |
| --- | --- | --- | --- | --- | --- |
| CT | ICA | ECA | MIX | Total | P |
| Unable to judge | 1 | 0 | 0 | 1 (3.8%) | 0.007 |
| ICA | 1 | 0 | 1 | 2 (7.7%) |  |
| ECA | 1 | 5 | 5 | 11 (42.3%) |  |
| MIX | 0 | 1 | 11 | 12 (46.2%) |  |
| Total | 3 (11.5%) | 6 (23.1%) | 17 (65.4%) | 26 |  |

SMI, superb microvascular imaging; ECA, external carotid artery; ICA, internal carotid artery; CT, computed tomography
